# Emergence of new phylogenetic lineage of Influenza D virus with broad antigenicity in California, United States

**DOI:** 10.1080/22221751.2021.1910078

**Published:** 2021-04-09

**Authors:** Chen Huang, Jieshi Yu, Ben M. Hause, Jie Yeun Park, Chithra Sreenivasan, Tirth Uprety, Zizhang Sheng, Dan Wang, Feng Li

**Affiliations:** a University of Kentucky, Lexington, KY, USA; b Cambridge Technologies, Worthington, MN, USA;; c Columbia University, New York, NY, USA

**Keywords:** Influenza D, genetic lineage, antigenicity, hemagglutinin-esterase fusion, variation

## Abstract

Influenza D virus (IDV), with bovines as a primary host, circulates widely in cattle populations across North America and Eurasia. Here we report the identification of a novel IDV group with broad antigenicity in U.S. bovine herds, which is genetically different from previously known lineages of IDV.

Influenza D virus (IDV) is a newly identified member of the family *Orthomyxoviridae*. Since its first identification in the United States, IDV and its antibody prevalence have been reported in North America, Europe, Asia, and Africa [1–3]. IDV utilizes cattle as a primary reservoir with a broad host range. To date, IDV isolations are largely from cattle and to some extent, from pigs, while IDV RNA genomes and/or specific antibodies have been detected in multiple animal species including goats, sheep, horses, buffalo, camels and wild boars [1–3]. IDV infection causes mild respiratory disease in cattle [4] and is associated with the bovine respiratory disease (BRD) complex [5–7] with a major economic impact to the cattle industry. Importantly, antibodies against IDV have also been reported in humans, especially those previously exposed to cattle [8,9].

Based on the nucleotide sequences of the Hemagglutinin-Esterase Fusion (HEF) protein that is the primary target of virus-neutralizing antibodies, IDV can be classified into four distinctive lineages, D/OK, D/660, D/Yama2016, and D/Yama2019 [10,11]. Here, we report the isolation of three novel influenza D viruses from diseased bovines with respiratory disease in California, United States. Characterization of the genome sequences and antigenic properties found that the three isolates formed a distinctive lineage, designated D/CA2019, which is genetically distinct from previously known IDV lineages. Significantly, the novel lineage exhibited broader antigenicity when compared to IDV D/OK and D/660 lineages that have widely spread through U.S. bovine population.

In this study, total 100 nasal swabs were collected by the herd veterinarian from calves exhibiting respiratory disease at commercial operations in 2018–2019. Of them, 10 samples tested positive for IDV in RT–PCR assay (10%, 10/100) were inoculated individually onto human rectal tumour HRT-18G cells, then subsequently passaged to swine testicle ST cells. When a cytopathic effect (CPE) was observed in ST cells, supernatants were collected and tested for the presence of hemagglutination (HA) using 0.5% of turkey red blood cells. Following successful virus isolation and initial genome sequencing, six of them were further employed for full-genome sequencing by MiSeq. Sequences were submitted to GenBank: D/bovine/Michigan/0377/2019 (D/MI/0377/2019) (MW632171-MW632177), D/bovine/California/1490/2018 (D/CA/1490/2018) (MW632178-MW632184), D/bovine/Oklahoma/1544/2018 (D/OK/1544/2018) (MW632185-MW632191), D/bovine/California/0363/2019 (D/CA/19) (MW020305-MW020311), D/bovine/California/1908/2019 (D/CA/1908/2019) (MW020319-MW020325) and D/bovine/California/0894/2019 (D/CA/0894/2019) (MW020312-MW020318).

To examine the evolutionary relationship of these new isolates to previously characterized IDVs, we performed phylogenetic analysis of the HEF segment sequences in MEGA-X, together with a total of 61genome sequences deposited in GenBank from 2013 to 2020. Three of six new isolates clustered together, forming a lineage that is genetically distinguishable from all the known IDV strains (Figure 1(A)). The novel group is provisionally designated IDV D/CA2019 lineage. Other three isolates belong to D/660 and D/OK lineages, respectively. The pairwise alignment of the HEF amino acid sequence of one representative strain of the D/CA2019 lineage with its counterpart in representative strains of four IDV lineages (D/OK, D/660, D/Yama2016, and D/Yama2019) revealed that D/CA2019 exhibited lower sequence homology with each of the previously identified lineages (94.58%–95.63%) (Figure 1(B)). The phylogenetic trees were also generated for other genome segments of D/CA2019 lineage isolates. Segments of D/CA2019 isolates were clustered together (Figure S1). The segments 1–2, and 5–7 of D/CA2019 isolates were phylogenetically close to representative strain D/bovine/Oklahoma/660/2013 (D/660/13) of D/660 lineage (Figure S1A,B, D–F). Nevertheless, their segment 3 grouped with D/swine/Oklahoma/1334/2011 (D/OK/11) strain, a representative strain of D/OK lineage (Figure S1C).

**Figure 1.**
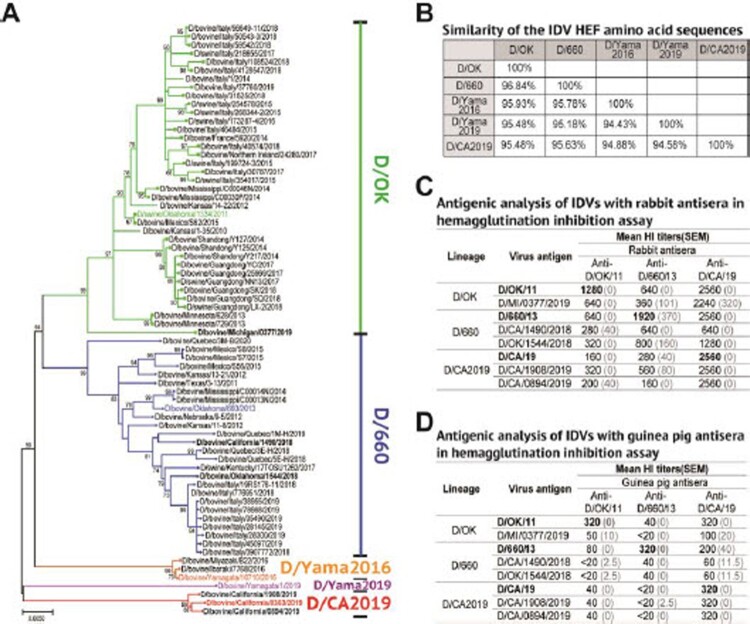
D/CA2019 is genetically and antigenically distinct from previously known lineages of IDV. (A) Maximum-likelihood phylogenetic tree for the HEF segments of IDVs. Nucleotide sequences of the HEF segment were aligned and analysed using MEGA-X, with 1000 bootstrap replicates. Bootstrap scores of at least 50 were shown to the left of the major nodes. Scale bar represents the number of substitutions per site. The branch of three D/CA2019 isolates from California was indicated in red colour. Lineages and their representative strains were labelled with different colours. New IDV isolates used in this study were bolded. (B) Similarities of amino acid sequences of the IDV HEF gene were analysed in the DNAMAN_8 software. Antigenecity of D/CA2019-lineage viruses was analysed by using rabbit (C) and guinea pig (D) antisera in HI assay. HI titres were mean titres from four independent experiments. Standard errors of the mean (SEM) were shown in parentheses.

To characterize the antigenic properties of new IDV isolates and further elucidate whether genetic divergence observed in D/CA2019 group correlates to antigenic variation, we performed a hemagglutination inhibition (HI) assay using rabbit reference antisera raised against semi-purified virus particle preparations from three IDV lineage-representative strains, D/OK/11, D/660/13, and D/CA/19. As summarized in Figure 1(C), the antiserum for the D/CA/19 strain reacted to itself (homologous) as well as to other two new isolates equally with an HI titre of 2560. Interestingly, the antiserum generated against the D/CA/19 strain also effectively recognized D/OK and D/660 lineages with HI titres of 640–2560. In contrast, the rabbit antisera raised against D/OK/11 or D/660/13 virus inefficiently recognized IDV D/CA2019 lineage viruses, with titres 4–8 fold lower than the homologous titre against D/OK/11 and with titres 3.4–12 fold lower than the homologous titre against D/660/13, respectively. These results clearly indicate that three D/CA2019 lineage isolates are antigenically different from D/OK and D/660 lineages, which correlate with the genetic variation observed among these viruses (Figure 1(A)). Our data also reveal that new IDV D/CA2019 lineage exhibits broad antigenicity, which may enable this group of viruses to elicit antibodies that can broadly neutralize diverse strains of IDV.

Antigenic variation between D/CA2019 and other two lineages was also further demonstrated in the HI assay using post-infection guinea antisera raised against three cell culture-propagated lineage-representative viruses as described above (Figure 1(C)). As summarized in Figure 1(D), IDVs in D/OK lineage were well recognized by the guinea pig antisera raised against the D/CA/19 virus at HI titres of 100–320, which were identical or higher when compared to the lineage-homologous titres for viruses in D/OK (HI titres: 50–320) lineage. IDVs in D/660 lineage were also readily reactive to the D/CA/19 guinea pig antisera with HI a titre of 200 for D/660/13 virus or 60 for other two viruses in D/660 lineage, which was slightly lower (within 2-fold) when compared to the homologous HI titre for D/660/13 virus (HI titre: 320) but was relatively higher than the lineage-homologous HI titre for other two viruses (HI titre: 40) within D/660 lineage. IDVs in D/CA2019 lineage were efficiently recognized by the guinea pig anti-D/CA/19 sera at HI titres of 320. Nevertheless, all three IDV/CA2019 lineage viruses were poorly recognized by the guinea pig antiserum raised against D/OK/11 and D/660/13 viruses, with titres at least 8-fold lower than the homologous titre of anti-D/OK/11 or anti-D/660/13 sera, respectively (Figure 1(D)). Antibody cross reactivity in guinea pigs further support our conclusion that D/CA2019 represents a novel lineage that is genetically and antigenically different from D/OK and D/660 lineages.

In summary, we identified a novel phylogenetic group of IDV strains in diseased bovines in California. This new group, D/CA2019, represents the fifth genetic lineage of IDV (Figure 1(A)). The anti-D/OK/11 and anti-D/660/13 sera had limited cross-reactivity to the viruses in the D/CA2019 lineage but the anti-D/CA/19 sera had broad cross-reactivity to multiple viruses in the other two lineages (Figure 1(C,D)) currently circulating in the U.S. bovine and pig populations, which indicats that D/CA2019 may evade the immune responses that were mounted previously against D/OK and D/660 lineages. In addition, contemporary strains representative of D/OK and D/660 lineages and their respective reference antisera are probably needed in future influenza D virus research because new isolates of D/OK or D/660 lineage appeared to diverge substantially in HI-based antigenicity assay from the past lineage-representative strain D/OK/11 or D/660/13 (Figure 1(C,D)). Overall, our new finding highlights a critical need for future surveillance of D/CA2019 lineage-like viruses in the U.S. agricultural animals toward better understanding the spread and pathogenicity of this novel IDV lineage.

## Supplementary Material

ChenFig_S1.tif

D-CA-MS_02152021_EMI_Suppl_.docx
