# Supplementary figures and images for: Emergence of new phylogenetic lineage of Influenza D virus with broad antigenicity in California, United States
Source: Emerg Microbes Infect. 2021 Apr 9;10(1):739–42. doi: 10.1080/22221751.2021.1910078 (PMC8043534; doi:10.1080/22221751.2021.1910078)

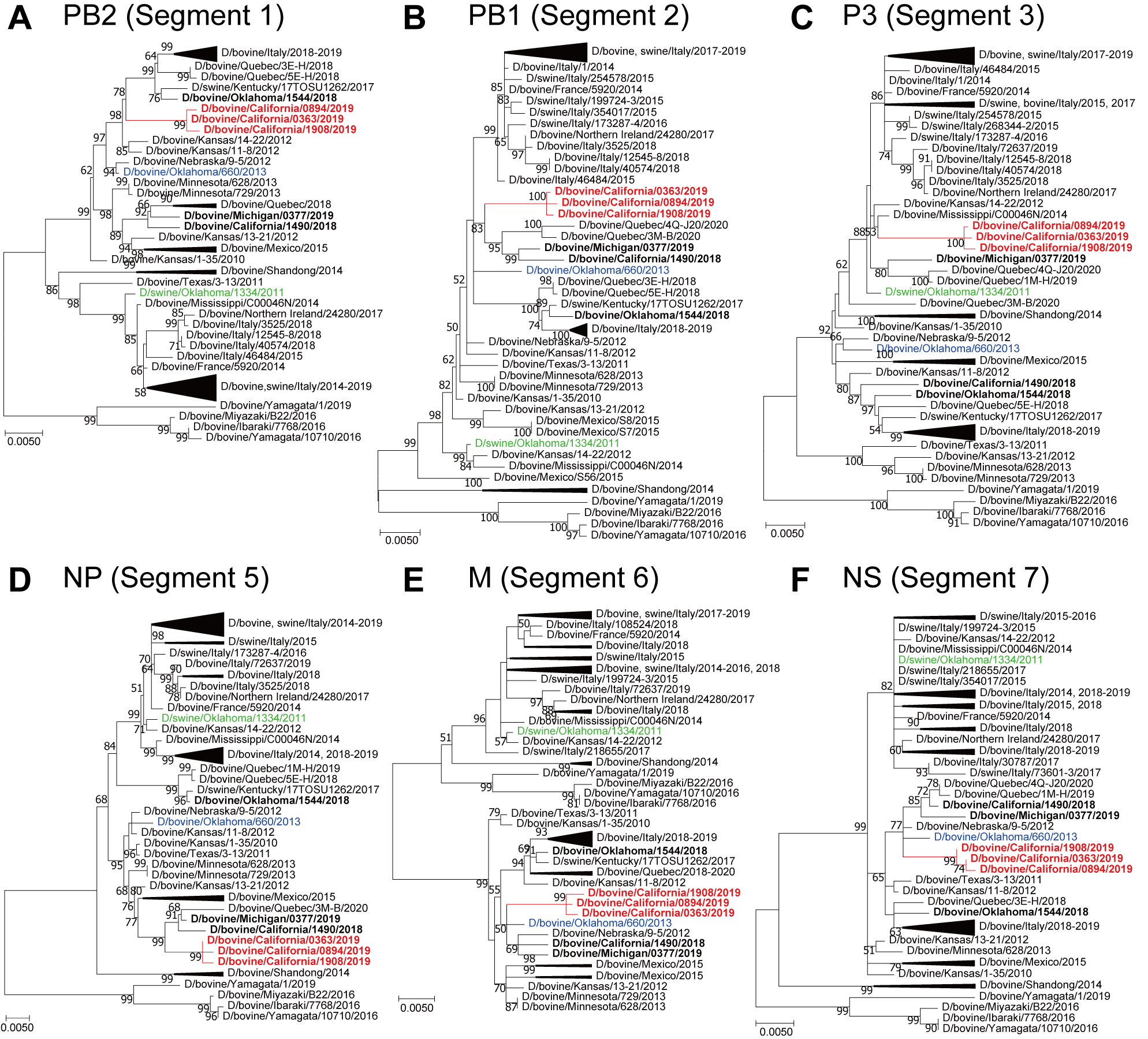

Supplement: ChenFig_S1.tif [file TEMI_A_1910078_SM9669.tif]
