# Supplementary material for: Emergence of new phylogenetic lineage of Influenza D virus with broad antigenicity in California, United States
Source: Emerg Microbes Infect. 2021 Apr 9;10(1):739–42. doi: 10.1080/22221751.2021.1910078 (PMC8043534; doi:10.1080/22221751.2021.1910078)
Supplement: D-CA-MS_02152021_EMI_Suppl_.docx [file TEMI_A_1910078_SM9668.docx]

**Figure S1. Phylogenetic trees for non-HEF segments of IDVs.** (A-F) In MEGA X, maximum-likelihood analysis in combination with 1000 bootstrap replicates was used to generate trees based on the nucleotide sequences of the segments 1 and 2 (Polymerase basic subunits 2 and 1, PB2 and PB1), segment 3 (polymerase subunit 3, P3), segment 5 (nucleoprotein, NP), segment 6 (matrix protein, M), and segment 7 (non-structural protein, NS). Bootstrap scores of at least 50 were shown to the left of the major nodes. Scale bar represents the number of substitutions per site. The branch of three D/CA2019 isolates from California was indicated in red color. Lineage representative IDV strains: D/OK lineage D/swine/OK/1334/2011 (D/OK/11), D/660 lineage D/bovine/660/2013 (D/660/13), and D/CA lineage D/bovine/California/0363/2019 (D/CA/19) were labeled with green, blue, and red colors, respectively. New IDV isolates in this study were bolded. Black triangles indicate compressed branches with virus strains isolated in the same locations.
